# Predicting self-harm in prisoners: Risk factors and a prognostic model in a cohort of 542 prison entrants

**DOI:** 10.1192/j.eurpsy.2020.40

**Published:** 2020-04-28

**Authors:** Howard Ryland, Charlotte Gould, Tristan McGeorge, Keith Hawton, Seena Fazel

**Affiliations:** 1 Department of Psychiatry, University of Oxford, Oxford, United Kingdom; 2 Central and North West London NHS Foundation Trust; 3 North Middlesex University Hospital NHS Trust

**Keywords:** Prediction, prison, risk factors, screening, self-harm, prediction model

## Abstract

**Background.:**

Self-harm is common in prisoners. There is an association between self-harm in prisoners and subsequent suicide, both within prison and on release. The aim of this study is to develop and evaluate a prediction model to identify male prisoners at high risk of self-harm.

**Methods.:**

We developed an 11-item screening model, based on risk factors identified from the literature. This screen was administered to 542 prisoners within 7 days of arrival in two male prisons in England. Participants were followed up for 6 months to identify those who subsequently self-harmed in prison. Analysis was conducted using Cox proportional hazard regression. Discrimination and calibration were determined for the model. The model was subsequently optimized using multivariable analysis, weighting variables, and dropping poorly performing items.

**Results.:**

Seventeen (3.1%) of the participants self-harmed during follow up (median 53 days). The strongest risk factors were previous self-harm in prison (adjusted hazard ratio [aHR] = 9.3 [95% CI: 3.3–16.6]) and current suicidal ideation (aHR = 7.6 [2.1–27.4]). As a continuous score, a one-point increase in the suicide screen was significantly associated with self-harm (HR = 1.4, 1.1–1.7). At the prespecified cut off score of 5, the screening model was associated with an area under the curve (AUC) of 0.66 (0.53–0.79), with poor calibration. The optimized model saw two items dropped from the original screening tool, weighting of risk factors based on a multivariable model, and an AUC of 0.84 (0.76–0.92).

**Conclusions.:**

Further work is necessary to clarify the association between risk factors and self-harm in prison. Despite good face validity, current screening tools for self-harm need validation in new prison samples.

## Introduction

Self-harm represents a substantial burden of morbidity in prisons. In England and Wales, rates of self-harm have increased sharply in recent years from 264 per 1,000 prisoners in 2004 to 629 per 1,000 prisoners in 2018, equivalent to 52,814 incidents [1]. An estimated 5–6% of male prisoners self-harm every year, with annual rates of 20–24% observed in female prisoners [2]. Of those who self-harm, around 6–7% of incidents are severe enough to require transfer to external hospitals [1]. Among the adverse outcomes following self-harm, the most serious is completed suicide inside custody and on release. In England and Wales, the mean annual rate for suicides in male prisoners who had self-harmed was 334 per 100,000, compared with 95 per 100,000 for those who had not self-harmed [2]. On release, there is a doubling of the odds of completing suicide in people who have self-harmed in prison, compared to other prisoners [3]. In addition, there is an eightfold increase in the rate of near-lethal suicide attempts following self-harm in prison [4]. Repetition of self-harm is common in prison, with male prisoners who self-harm doing so on average around twice per year, while female prisoners repeat self-harm around eight times per year [2]. Risk factors for self-harm can be divided into acute or triggering factors and predisposing factors. In terms of acute factors, the evidence shows that being imprisoned for fewer than 30 days, being on remand, single-cell accommodation and being intimidated to hand over belongings are important. For predisposing factors, psychiatric disorders have been shown to be replicated risk factors. Psychosocial risk factors include having a family member who died by suicide and having no close friends outside of prison [4–6].

The extent of self-harm in prison, which represents considerable physical and psychological morbidity in prisoners,  also leads to high levels of stress and potential burnout in prison staff [7]. At the same time, the prison workforce is reported to be under increasing pressure to manage self-harming behavior [8]. Managing those prisoners who repeatedly self-harm can put particular pressure on the system, including high levels of frustration, tension between staff groups, and a lack of job control and agency for prison officers [7]. The combination of a high prevalence of self-harm and limited workforce capacity make a brief, effective screening tool to identify those at high risk of self-harm on prison entry potentially useful for treatment allocation. In addition, a valid screen could assist in linking individuals who are at high risk of self-harming with mental health and substance misuse services [9]. Scalable risk predictions have been shown to be effective in the related context of secure psychiatric hospitals for the prediction of violence within hospital and on discharge [10, 11]. Current screening tools for suicide and self-harm in the prison context are limited by a number of factors, including not being developed for prisoners specifically and poor face validity [12]. We report the development and testing of a screening tool for self-harm in prisoners on arrival to custody.

## Methods

### Screening tool development

A short screening tool was developed from a review of the literature for validated risk factors from studies of near-lethal attempts [4] and a systematic review of suicide in prison [13]. The tool contained 11 items and had separate versions for men and women based on research evidence for different risk factors for suicidal outcomes in male and female prisoners. The questions covered a range of historical, clinical, social, and prison-related factors. Each item had a binary response (yes/no). In model development, we opted for an unweighted model as we thought that a simple model would be more feasible in prisons, which could be completed on paper records. Each question therefore corresponded to a score of 1 or 0. A predefined cut-off score was determined as 5 or above denoting high risk based on testing the scales in a near-lethal sample [14]. For the purposes of this report, the screening tool is called Oxford Self-Harm in Prison (OxSHIP). This report is based on the male version.

### Participants

The instrument was tested at two male prisons in England to provide a range of prison settings where new arrivals could be screened. A protocol was developed, not previously published but included in Supplementary Materials. Her Majesty’s Prison (HMP) Wormwood Scrubs in London is a local category B prison (B is the second highest security category in England and Wales from A to D) [15]. HMP Woodhill in Milton Keynes has a dual role as a local prison and also holds category A prisoners (the highest security category of prisons) [16]. The tool was administered in addition to the current systems in place for detecting and managing prisoners at high risk of suicide and self-harm (which involve a single question about whether a prisoner feels like self-harming or attempting suicide in the health screen on arrival). Where prisoners made direct statements while the tool was being administered of intent to self-harm or end their lives, this was communicated to staff to ensure that appropriate steps were taken to manage risk, as per the research protocol [17]. These individuals remained in the study cohort.

The tool was administered to newly arriving prisoners at each prison by one of the authors (C.G.) within 7 days of arrival. New receptions (i.e., those coming directly from the community as opposed to those being transferred from another prison or returning to prison from court) were purposively selected. It was hypothesized that those who are newly entering the prison system would be at an increased risk and this approach allowed the study to be targeted toward a defined population [18–20]. Prison officers provided newly arriving prisoners with information sheets about the study. The researcher was supplied with a list of new prisoners from prison staff. Those prisoners meeting the inclusion criteria were approached by the researcher on the reception, detoxification, and main prison wings to advertise the study individually to potential participants. Participants were selected via convenience sampling and were usually initially approached within 24 h of arrival. The number of potential participants was often in excess of what could be screened within the agreed time frame. Information on those who refused was not collected, as refusals occurred at different stages. Some refusals occurred before being approached by the researcher, and we did not have ethics approval to collect information about these individuals. Additionally, structural factors, such as prison lock downs (where prisoners cannot leave their cells) and unavailability of staff to escort prisoners, prevented accurate recording of the characteristics of eligible individuals who did not participate. For these reasons, it was also not possible to reliably calculate the participation rate.

Based on an estimate of the level of self-harm in male prisoners in England and Wales of 5% [2], a power calculation suggested that the tool needed to be administered to 540 prisoners to differentiate a 1-unit mean difference in instrument scores between those who self-harmed and those who did not, at an alpha of 0.05 and a beta of 0.8.

### Data collection

Self-harm was identified through a number of ways, including self-report by prisoners, concerns raised by other prisoners and through investigation of observed injuries by prison officers. A specific department within the prison (called the Safer Custody team) was notified of each incident and a risk management plan initiated. Incidents of self-harm were recorded according to an established protocol, which includes the documentation of the injury and any treatment, using a standardized form. Hunger strikes or food refusal are not considered self-harm according to the prison service in England and Wales.

Follow-up information on self-harm incidents was collected from Safer Custody teams at both prisons. These teams are responsible for the management of prisoners who are at risk of harm to self, to others, and from others [21]. The primary objective was to assess the tool for the probability of self-harm within 6 months of initial screening. Her Majesty’s Prison and Probation Service (HMPPS) define self-harm as “any act where a prisoner deliberately harms themselves irrespective of the method, intent or severity of any injury” in which no underlying assumptions of intent or motivation are made [20]. The first episode of self-harm in a particular individual was defined as the outcome.

### Ethical approval

The study was approved by the National Research Ethics Service (NRES) Committee London—South East (REC reference 15/LO/1235) on August 25, 2015. The authors assert that all procedures contributing to this work comply with the ethical standards of the relevant national and institutional committees on human experimentation and with the Helsinki Declaration of 1975, as revised in 2008.

### Statistical analysis

Statistical analysis was conducted using Cox proportional hazard regression, adjusting for the score at screening [22]. No other variables were included in the model. The prespecified cut-off for low versus high risk was <5 versus ≥5. Items scored as “do not know” were recoded as “0.” This was consistent with how overall scores were previously derived. There were no missing data for individual item scores. The “at-risk” period spanned from the interview date until self-harm, transfer, release, or end of follow-up (6 months post-interview). Sensitivity, specificity, and positive and negative predictive values were calculated using the prespecified cut-off score of five or above [14,23]. Discrimination was summarized using the area under the curve (AUC) and calibration with Brier score. The latter, which is the proportion of predicted and observed events at different screening scores, was compared using a calibration plot [24]. The Brier score is not informative on its own but should be compared with the Brier score at 0 and the mean. If the model’s Brier score is lower than these two values, this would indicate good calibration [25].

In secondary analyses, which we planned after the data were collected, we sought to improve statistical measures of predictive performance by (a) varying cut-offs for low versus high risk, (b) creating a multivariable model with each item weighted separately, and (c) additionally excluding some items from the multivariate model.

This process of optimization involved four steps:

1. The first step was to consider the total score as a continuous variable, with a score from 0 to 11. Each one-point increase in the total score had the same relative effect on the predicted outcome, which would no longer be restricted to high or low, but had 12 different risk categories. There was a weighting assigned to the total score. The AUC was determined across all 12 risk categories.
2. The second step was to consider other discrete cut-off scores other than 5 to determine the model performance as a binary tool at each alternative level.
3. The third step was to consider each item separately and assign each its own weighting. This resulted in 2,048 (211) possible predicted outcomes. In this scenario, the AUC should be seen as a summary measure across all 2,048 risk categories, although in practice there were far fewer as not all possible ways of scoring were represented in the study cohort.
4. The final step was to repeat the third step but remove some items from the model to determine the effect on the AUC. This selection of items for removal was guided by statistical significance (set at *p* < 0.05).

All analyses were conducted using STATA (Version 11.2).

## Results

### Sample

A total of 542 male prisoners completed the screening tool. Of these, 364 participants were recruited at HMP Wormwood Scrubs between November 11, 2015 and May 5, 2016 and 178 participants at HMP Woodhill between May 10, 2016 and November 21, 2016. One hundred eighty-nine (34.9%) participants were completely new to custody. Seventeen (3.1%) of the total sample went on to harm themselves within 6 months. The median follow-up was 53 days (interquartile range: 22–118 days). There was no loss to follow up from the consented cohort.

### Demographics

Mean age was 33.2 years old, with a median age of 31 years (range 18–81). Most identified as White British (*n* = 224, 41.4%). A total of 311 (57.6%) were single and 61 (11.3%) were married. On average, participants had left education at the age of 17.3 years, with a median of 16 years. In total, 86 (15.9%) were unemployed and 187 (34.6%) were working full time at the time of prison entry. The largest proportion were living with friends and family (*n* = 164, 30.4%). Of them, 144 (26.7%) were undergoing substance detoxification, 251 (53.5%) were on remand, of whom 85 (29.5%) were charged with committing a violent offence. For sentenced prisoners, the average length of sentence was 20.9 weeks and 33 (13.1%) were convicted of violence against the person (Table 1 and Tables S1 and S2).

**Table 1.** Demographic characteristics of 542 male prisoners screened on arrival

Characteristics	*N*	%
Ethnicity		
White British	224	41.4
White other	82	15.2
Black British	46	8.5
Black African	54	10.0
Black Caribbean	10	1.9
Asian	13	2.4
Asian British	50	9.2
Mixed/multiple ethnicity	48	8.9
Other	14	2.6
Relationship status		
Single	311	57.6
Married	61	11.3
Long term relationship/co-habiting	141	26.1
Separated, divorced, or widowed	27	5.0
Employment status		
Unemployed	86	15.9
Full-time	187	34.6
Part-time	40	7.4
Registered sick or disabled	83	15.4
Claiming benefits	58	10.7
Self-employed	36	6.7
Retired or student	25	4.6
Other	25	4.6
Housing status		
Friends/family	164	30.4
Private rented	129	23.9
Hostel	34	6.3
Sleeping rough	69	12.8
No fixed abode (NFA)	14	2.6
Council/housing association	91	16.9
Homeowner	36	6.7
Other	3	0.6
Substance detoxification		
Yes	144	26.7
No	395	73.3
Offending information		
Previous period in prison		
Yes	363	67.2
No	177	32.8
Recall to prison		
Yes	72	13.3
No	468	86.7
Current remand status		
Yes	289	53.5
No	251	46.5

Note: missing data for ethnicity *n* = 1; for relationship, employment, housing, and offending *n* = 2 and for substance detoxification *n* = 3.

### Risk factors

In multivariable analyses, hazard ratios (HRs) for each item in the screen varied from 0.3 to 9.3 (Table 2). The strongest risk factors in the multivariable model were self-harm in prison (adjusted hazard ratio [aHR] = 9.3 [3.3–26.6]) and current suicidal ideas (aHR = 7.6 [2.1–27.4]) (Table 2). As a continuous score, a one-point increase in the screen was significantly associated with self-harm (HR = 1.4, 1.1–1.7).

**Table 2.** Risk factors for self-harm in 542 male prisoners in the first 6 months after entering prison.

Number	Question	Number scoring positively^*^	Percentage scoring positively^*^	Univariable hazard ratio (95% CI)	Multivariable hazard ratio (95% CI)
1	Are you currently diagnosed with an emotional or psychiatric disorder or are you currently being prescribed medication for emotional, psychological or psychiatric problems?	172	31.9	1.6 (0.6–4.4)	0.4 (0.1–1.6)
2	Have you ever had psychiatric treatment by a medical health professional?	205	38.0	2.1 (0.8–5.3)	2.0 (0.6–7.2)
3	Have you ever attempted suicide or self-harmed outside prison?	135	25.0	2.6 (1.0–6.8)	1.3 (0.4–4.5)
4	Have you ever attempted suicide or self-harmed inside prison?	55	10.2	11.0 (4.3–28.7)	9.3 (3.3–26.6)
5	Do you have current thoughts about wanting to harm yourself?	33	6.1	6.5 (2.3–18.5)	7.6 (2.1–27.4)
6	Have any of your family died by suicide or self-harmed themselves?	117	21.7	0.8 (0.2–2.7)	0.3 (0.1–1.3)
7	Do you have friends or family that you feel close to?	76	14.1	0.9 (0.2–3.9)	0.7 (0.1–3.1)
8	Have you experienced homelessness in the past for a month or more?	209	38.8	2.7 (1.0–7.0)	2.4 (0.8–7.2)
9	Were you ever in local authority care before the age of 16?	110	20.4	2.3 (0.9–6.2)	2.2 (0.7–6.7)
10	Have you been in prison before?	353	65.4	1.3 (0.4–3.6)	0.6 (0.2–1.9)
11	Do you feel that the future is hopeless and that things cannot improve?	58	11.0	1.2 (0.3–5.1)	0.4 (0.1–2.2)

Abbreviation: CI, confidence interval.

* For question 7, yes scored 0 and no scored 1. For all other questions, yes scored 1 and no scored 0. All items in the multivariable model are adjusted for simultaneously.

### Performance of the unmodified screening tool

**Figure 1.**
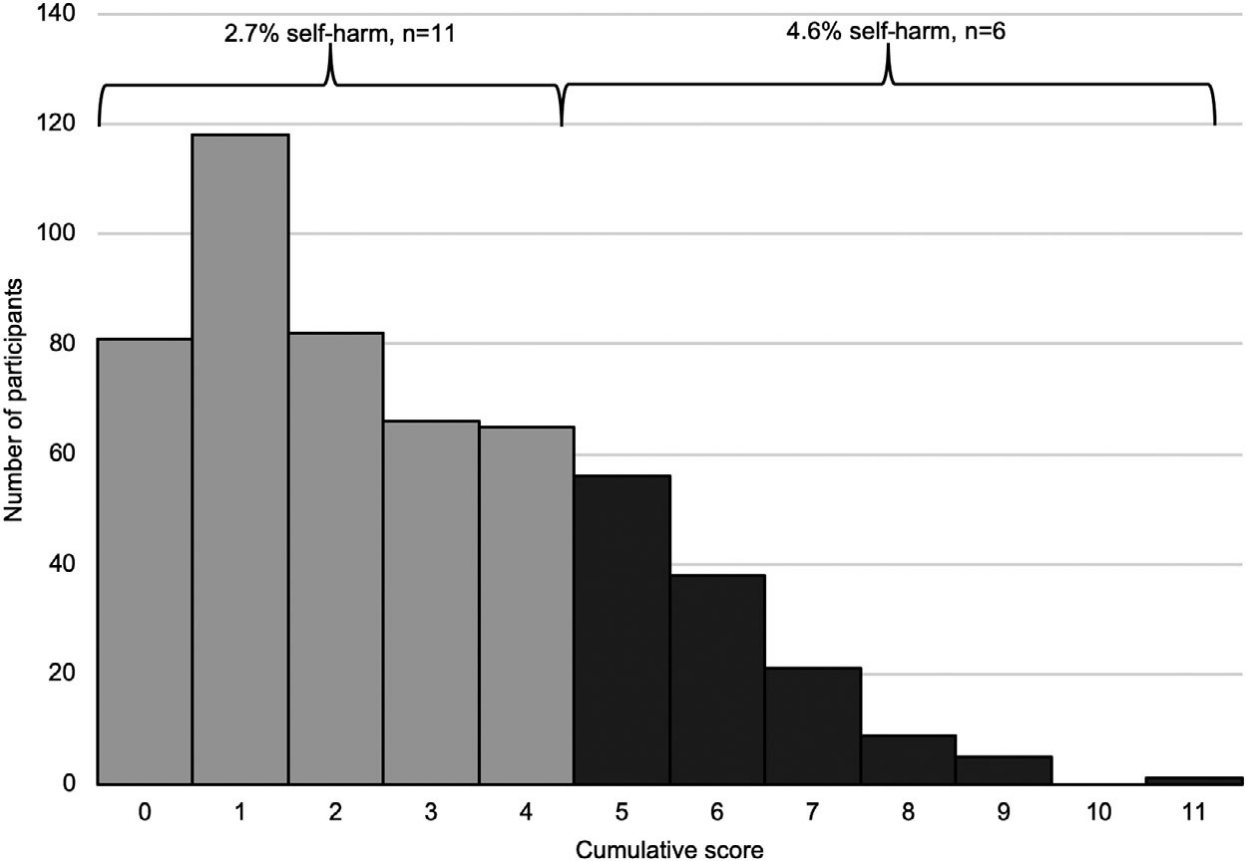
Histogram of participants by cumulative score on the screening tool.

Using the prespecified cut off of 5, of those with a score of 4 and lower (412/542 or 76.0%), 11 (2.7%) went on to self-harm. Of those with score of 5 and higher (130/542 or 34.0%), 6 (4.6%) went on to self-harm (Figure 1). This represents a sensitivity of 35% (14–64%), specificity of 76% (73–80%), positive predictive value (PPV) of 5% (2–9%), and negative predictive value (NPV) of 97% (96–98%) (Table S3).

An analysis of the area under the receiver operating characteristics curve for each cut-off gave an AUC score of 0.66 (95% CI: 0.53–0.79), indicating at best moderate performance (Figure S1) [26].

Observed risk was plotted against predicted risk and a Brier score of calibration calculated. The Brier score for the model was 0.0316, with Brier_0_ 0.0314 and Brier_mean_ 0.0314, indicating poor calibration of the model (Figure S2) [25].

### Secondary analyses

The performance of the tool was examined for each different cumulative score. With a cut-off score of 4, the sensitivity increased from 35 to 59%, while specificity dropped slightly from 76 to 65%, with PPV and NPV almost unaffected. If the cut off was raised to 6, specificity increased from 76 to 87%, with no effect on sensitivity and an increase in PPV from 5 to 8% (Table S4).

When we conducted multivariable modeling, items 3 (Have you ever attempted suicide or self-harmed outside prison?) and 7 (Do you have friends or family that you feel close to?—scored as 1 if the response was negative) were not associated with the outcome, and were then dropped to improve performance (see multivariable HRs in Table 2). The resultant AUC (based on weighting the remaining nine predictors) increased from 0.66 (0.53–0.79) to 0.84 (0.76–0.92) for the new model (Table S5).

## Discussion

### Main findings

This study presents the evaluation of a model to predict self-harm in 542 male prisoners. As part of this, we prospectively examined a range of demographic, clinical, and social risk factors that had been previously identified in studies of suicide and near-lethal attempts in prisoners. In multivariable analyses, we found that the strongest associations with self-harm in the first 6 months of prison entry were previous self-harm inside prison (aHR 9.3 [95% CI: 3.3–26.6]) and current thoughts of self-harm (aHR 7.6 [2.1–27.4]). There were also nonsignificant associations with recent homelessness and a history of childhood local authority care. We also tested the performance of these risk factors as part of an unweighted 11-item screening tool for self-harm, which was subsequently optimized to a weighted 9-item instrument.

The overall discrimination of the original 11-item screen was moderate at best (AUC = 0.66 [0.53–0.79]) [27]. At the prespecified cut off of 5, the sensitivity was low, specificity moderate, positive predictive value very low, and negative predictive value high. This may be partly explained by a low number of events, although these were in line with the expected rates of self-harm. Based on a rate of 5% in a year, we estimated 13–14 prisoners to self-harm within 6 months, compared to the 17 observed [2]. The Brier score indicated poor calibration, which means that using the screen as an actuarial score will lack accuracy [25].

In order to explore ways that the model could be improved, we undertook a number of post hoc adjustments that were deviations from the protocol. The wide range of HRs for individual items in the univariable analysis suggested that weighting variables would improve performance. The high HRs associated with two items (previous suicide attempt or self-harm in prison and current thoughts of self-harm) further indicated that weighting variables according to effect size would be required to optimize predictive performance. Based on multivariable analyses, we weighted predictors and dropped some factors from the optimized model. The predictive power of this new model was materially improved from an AUC of 0.66 (0.53–0.79) to 0.84 (0.76–0.92) using the same dataset [27]. The latter AUC would indicate good to excellent discrimination.

### Implications for clinical practice

To examine generalisability, we compared selected demographics of the sample population to the overall prison population. The mean age of 33.2 was younger than 36.8 in the general adult prison population in England and Wales. The proportion of white prisoners was similar (56.6% vs. 58.6% in all prisoners). The sample included a higher proportion of prisoners on remand (53.5% vs. 11.1% in all prisoners), as both the recruiting prisons were those that receive people from local courts [28].

Self-harm is a serious and increasing problem in prisons [1]. There is no consensus about how to manage it most effectively. However, identification of those at high risk is a first step that needs to be accompanied with linkage to effective interventions. Questions have been raised about whether self-harm tools are any more effective than clinical judgment in picking up those at higher risk [29]. This study is a novel attempt to translate evidence from epidemiological data on risk factors to develop a practical screening tool and then test the model in a real-world sample. The unmodified version of the screening tool had moderate discrimination and poor calibration, and therefore should not be used to identify those at high risk. The screening tool’s high negative predictive value may indicate limited potential for use as a way of screening out individuals at low risk of self-harm. In the absence of current validated tools, safety planning for all prisoners should be considered that includes restriction of means for self-harm and suicide, assessment and treatment of mental health problems, and providing all prisoners with information on how to access support including from clinical services, prison officers, Samaritans, their own social networks, and other prisoners. Future research can consider the performance of tools for subgroups inside prison at higher risk, such as those incarcerated for the first time. An alternative approach, if first-time in prison is found to be an independent risk factor, is that this item is added as a predictor to a tool administered to all prisoners.

### Implications for the development of risk assessment tools

In secondary analyses, we deviated from the original protocol. This resulted in a new weighted, nine-item model with improved predictive accuracy. It is notable that the items that dropped out of the multivariable model were not those with the lowest HRs on univariable analysis demonstrating how univariable modeling may not identify predictors. Using the original derivation dataset, the subsequent transformations increased the AUC from 0.66 (indicating moderate discrimination) to 0.84 (suggestive of good discrimination). However, this approach is problematic, as the lack of a protocol for these secondary analyses means that the new model is likely to perform differently in new samples [30].

This underscores the importance of interpreting validation studies for risk assessment tools and prediction models with caution. It is important to test candidate predictors in multivariable analyses, and consider weighting factors for any tool, as individual items have varying magnitude of effects and can influence each other in unpredictable ways. Another challenge of risk assessment tools, if the event rate is low, is the prevention paradox where most events occur in the lowest risk category. This was the case for the unadjusted tool, where 11 out of the 17 events occurred in the low risk category (i.e., had a score less than 5 out of 11). This means that the tool would not correctly identify the majority of those individuals at high risk of self-harm, limiting its usefulness from a population health perspective in an unmodified form. Another challenge for risk assessment tools is shrinkage, whereby the adjusted model appears to have a much-improved AUC but will likely perform worse in a new sample. To mitigate against this problem, it is necessary to test derived models in a new sample that is different to the one in which it was developed.

### Strengths and limitations

The development of the original screening tool used a predefined protocol. The selection of the items for inclusion was based on careful review of the available literature. Items covered a range of both historical and current factors which included behavioral, medical, and social dimensions. The screen is short and easy to use, with a predefined cut off. The study was performed in the prison population of interest and the outcome clearly defined. Discrimination and calibration measures were determined and transparently reported [31].

An important limitation is that the original model was not adequately powered, and the low number of observed events created model instability. The power calculation was designed to detect differences in risk factors; however, it is recommended that there should be at least 10 events per variable in derivation studies for predictive models [32,33]. Although the size of the overall cohort was large at 524, it needed 110 self-harm episodes to be adequately powered. The original model neither weighted individual items, nor examined multivariable effects. Internal validation of the model by bootstrapping was not undertaken, due to the limited performance of the model.

The optimized risk prediction model attempted to redress these shortcomings; however, importantly it lacked a prespecified protocol. In addition, the low event rate did not provide sufficient information to reliably make choices about which items could be removed. The final nine-item tool showed an apparently improved performance in the derivation sample, but it would need to be tested in a suitably large new sample to determine real world performance. The likelihood is that it would perform worse than expected for the reasons outlined above [30,31].

All predictors in the screening tool were self-reported and were not validated from other sources (e.g., medical records). Future work could compare responses with other sources of information (e.g., medical records). Not all episodes of self-harm are recorded, although it is unknown what proportion are missed. Novel psychoactive substances are widely used in some prisons, although their effects on self-harm are not known. Their use in screening tools, however, is unlikely to be useful as their prevalence and pattern of use changes rapidly in prison populations. There may be additional benefits to identify those who have undertaken more serious self-harm episodes, as these are more clearly associated with future suicide [34]. They also lead to more costs and healthcare resources. Future tools could consider identifying the risk of two outcomes, any self-harm and severe self-harm. The current study was underpowered to do so. We were unable to study the version of the tool developed for women prisoners, due to small number of events, but we collected qualitative information about its acceptability and use, which will be reported separately.

## Conclusions

This study highlights some of the challenges for developing an effective screening tool for identifying prisoners at high risk of self-harm. It demonstrates the importance of adhering to methodological best practice to avoid a number of pitfalls that threaten to undermine the accuracy and applicability of prediction models inreal world settings. In addition, this investigation tested potentially important risk factors for self-harm in a prospective, multivariable model that can inform further model development. Future work in this area should follow guidelines for prediction modelling including careful selection of candidate variables, which should be tested in multivariable analyses and then externally validated in new samples [30]. In the absence of current valid screening tools for suicide risk, safety planning for all prisoners should be considered.

## Data Availability

The study protocol is available in the Supplementary Materials. Additional tabular data is available on request from the authors. Individual participant data cannot be shared, as determined by the granted ethical approval.

## Supplementary material

For supplementary material accompanying this paper visit http://dx.doi.org/10.1192/j.eurpsy.2020.40. click here to view supplementary material
