# Supplementary material for: Predicting self-harm in prisoners: Risk factors and a prognostic model in a cohort of 542 prison entrants
Source: Eur Psychiatry. 2020 Apr 28;63(1):e42. doi: 10.1192/j.eurpsy.2020.40 (PMC7242092; doi:10.1192/j.eurpsy.2020.40)
Supplement: Supplementary file 1 [file S0924933820000401sup001.pdf]

Other supplementary material

Supplemental Table 1: Index offence of prisoners on remand (N=289)

Offence	N	%
Violence against the person	85	29.5
Sexual offence	16	5.6
Drug offence	33	11.4
Other	154	53.5

Note: missing data=1

Supplemental Table 2: Index offence of convicted prisoners (N=251)

Offence	N	%
Violence against the person	33	13.1
Sexual offence	9	3.6
Robbery	13	5.2
Fraud and forgery	13	5.2
Other theft	66	26.3
Criminal damage	5	2.0
Drug offence	15	6.0
Other	80	31.9

Supplemental Table 3: Discrimination parameters and statistics of OxSHIP based on the original model

	Self-harm	No self-harm	Total
Score ≥5	6	124	130
Score <5	11	401	412
Total	17	525	542

Sensitivity: 35% (14-62%)
Specificity: 76% (73-80%)

PPV: 5% (2-9%)
NPV: 97% (96-98%)

Supplemental Table 4: Discrimination parameters and statistics of the original OxSHIP model based on all possible cut-off scores

Score	TP	TN	Sens	Spec	PPV	NPV
0+	17	0	100%	0%	3%	---
1+	17	80	94%	15%	3%	99%
2+	16	196	82%	37%	4%	98%
3+	14	276	71%	53%	5%	98%
4+	12	340	59%	65%	5%	98%
5+	10	401	35%	76%	5%	97%
6+	6	457	35%	87%	8%	98%
7+	6	493	24%	94%	11%	97%
8+	4	513	18%	98%	20%	97%
9+	3	521	12%	99%	33%	97%
10+	2	524	0%	100%	0%	97%
11	0	525	0%	100%	0%	97%

Supplemental Table 5: Coefficients for each item in the optimised model

	Question	Coefficient
1	Are you currently diagnosed with an emotional or psychiatric disorder or are you currently being prescribed medication for emotional, psychological or psychiatric problems?	-0.8785
2	Have you ever had psychiatric treatment by a medical health professional?	0.8368
4	Have you ever attempted suicide or self-harmed inside prison?	2.2428
5	Do you have current thoughts about wanting to harm yourself?	2.1095
6	Have any of your family died by suicide or self-harmed themselves?	-1.1086
8	Have you experienced homelessness in the past for a month or more?	0.8132
9	Were you ever in local authority care before the age of 16?	0.8529
10	Have you been in prison before?	-0.6072
11	Do you feel that the future is hopeless and that things cannot improve?	-0.8742

LC (linear combination)=∑ beta*value of risk factor
Risk of self-harm incident within 6 months = 1 – 0.9723^exp(LC)

Supplemental Figure 1: Area under the receiver operating characteristics curve (AUC) for the original screening tool

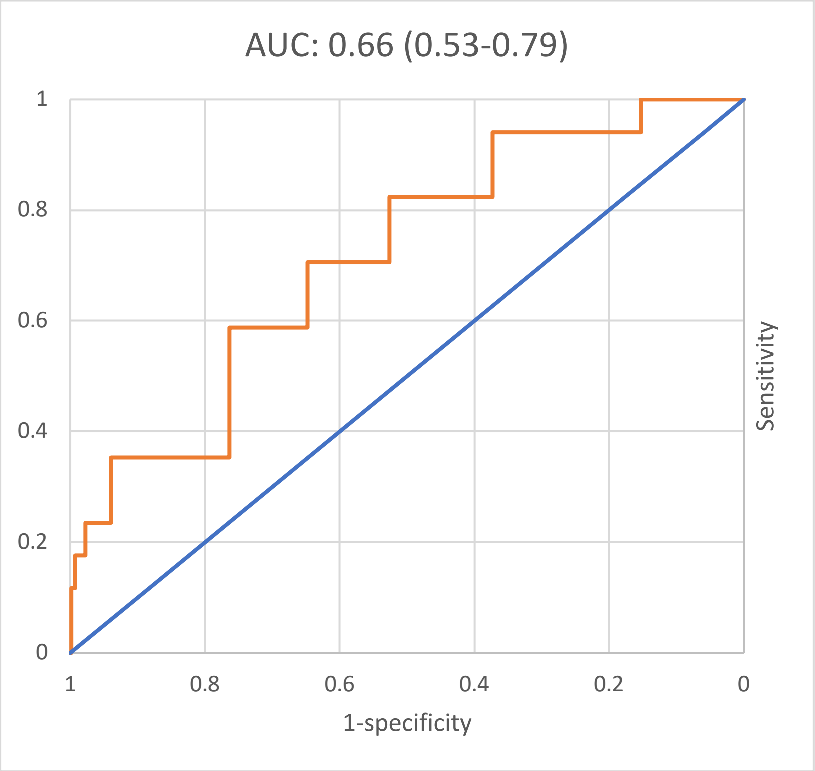

Supplemental Figure 2: Calibration plot for the original screening tool

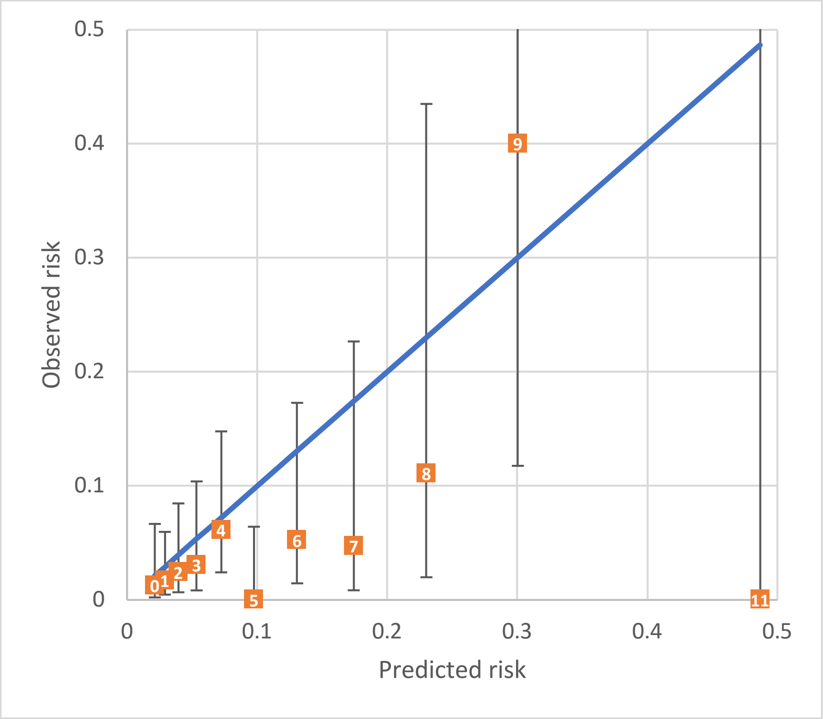

Clinical prediction rule for male prisoners for risk of self-harm – protocol

Study Summary

Design

Cohort study of prisoners in two prisons: HMP Wormwood Scrubs, HMP Woodhill. Prisoners were screened between Nov 11, 2015 and May 5, 2016 using an 11-item screening questionnaire (with a total score ranging from 0 to 11).

Outputs

The main objective is to assess a model that estimates the probability of self-harm within 6 months of screening, with appropriate measures of predictive accuracy, using the pre-specified risk cut-off.

Statistical Analysis

Statistical analysis will be based on Cox proportional hazard regression, adjusting for the score at screening. No other variables will be included in the model.
The pre-specified cut-off for low vs. high risk is <5 vs ≥ 5.
Items scored as 'D/K' will be recoded as '0'. This is consistent with how overall scores were previously derived. There is no missing data.
The 'at-risk' period will span from the interview date until self-harm, transfer, release, or end of follow-up (6 months post-interview). Re-entry after transfer or release will not be included.

Validation and goodness of fit

Sensitivity, specificity, and positive and negative predictive values will be calculated using the pre-specified cut-off.
Internal validation will be assessed using bootstrapping.
Predictive accuracy will be summarised using the concordance index and the Brier score.
The proportions of predicted and observed events at different screening scores will be compared using a calibration plot.

Screening questionnaire

#	Question	Yes/No
1	Are you currently diagnosed with an emotional or psychiatric disorder or are you currently being prescribed medication for emotional, psychological or psychiatric problems?	
2	Have you ever had psychiatric treatment by a medical health professional?	
3	Have you ever attempted suicide or self-harmed outside prison?	
4	Have you ever attempted suicide or self-harmed inside prison?	
5	Do you have current thoughts about wanting to harm yourself?	
6	Have any of your family died by suicide or self-harmed themselves?	
7	Do you have friends or family that you feel close to?	
8	Have you experienced homelessness in the past for a month or more?	
9	Were you ever in local authority care before the age of 16?	
10	Have you been in prison before?	
11	Do you feel that the future is hopeless and that things cannot improve?	
**Total score**		**/11**
